# Patient satisfaction after breast cancer surgery: A prospective clinical trial

**DOI:** 10.1007/s00508-020-01730-w

**Published:** 2020-09-03

**Authors:** Carmen Leser, Yen Y. Tan, Christian Singer, Robert Zeillinger, Florian Fitzal, Johann Lehrner, Daniel König, Christine Deutschmann, Daphne Gschwantler-Kaulich

**Affiliations:** 1 Department of Obstetrics and Gynaecology, Medical University of Vienna, Waehringer Guertel 18–20, 1090 Vienna, Austria; 2 Comprehensive Cancer Center, Medical University of Vienna, Vienna, Austria; 3 Department of Surgery, Medical University of Vienna, Vienna, Austria; 4 Department of Neurology, Medical University of Vienna, Vienna, Austria; 5 Clinical Division of Social Psychiatry, Department of Psychiatry and Psychotherapy, Medical University of Vienna, Vienna, Austria

**Keywords:** Breast cancer, Oncoplastic breast surgery, Quality of life, Breast analyzing tool, Cosmetic results

## Abstract

**Background:**

This study investigated the impact of curative breast cancer surgery on patient satisfaction concerning cosmetic results and quality of life (QoL).

**Methods:**

In this study 61 participants completed questionnaires to evaluate their QoL and patient satisfaction with cosmetic results following breast cancer surgery. Cosmetic outcomes were evaluated by the breast surgeon and an independent breast specialist using the Harris scale and the breast analyzing tool (BAT).

**Results:**

Of the participants 71% completed all 4 follow-up visits, 38 (62%) patients received breast-conserving therapy (BCT) and 23 (38%) received a mastectomy. Surgery-associated complications arose in 2.6% of the patients who received BCT and 17.4% of patients who received a mastectomy. No significant differences in QoL between BCT patients and mastectomy patients were observed immediately after surgery, or after 6 and 12 months. Breast asymmetry, measured using the BAT score, and QoL scores were worst immediately after surgery. The surgeon rated the cosmetic results as better compared to the independent breast expert (*p* = 0.001). Furthermore, patients aged over 60 years old were less satisfied with the cosmetic outcome compared to younger patients at the time of discharge (*p* = 0.024). Patients who received a mastectomy were less satisfied when the resected volume was higher.

**Conclusion:**

Patient satisfaction was lowest immediately after surgery but improved during the following months, despite continued breast asymmetry. For mastectomy patients, a lower resected volume led to a higher satisfaction with cosmetic results. Satisfaction is subjective and cannot be determined from the esthetic satisfaction of the surgeon or using an objective tool measuring breast asymmetry.

## Introduction

The female breast plays an important role in society and in the lives of individual women. Besides its physiological role in breastfeeding, it is culturally associated with womanhood and fertility and represents a prominent secondary sex characteristic [1, 2]. Therefore, women who have undergone breast cancer surgery may suffer not only from the sequelae of surgery, but may also feel compromised with respect to their femininity [3, 4]. A patient’s satisfaction with the surgical outcome is influenced not only by socioeconomic factors, ethnicity, and medical knowledge, but also by the surgical technique used, side effects of radiotherapy, and size or shape-associated asymmetry [5].

Interestingly, a patient’s satisfaction with the surgical outcome often differs considerably from the treating physician’s perception [6]. While some women are satisfied with the cosmetic outcome, the treating physician is not; conversely, the physician might be satisfied with the esthetic result, considering possible surgical difficulties, while the patient might have expected a better outcome [6].

Considering the type of breast surgery there are remarkable differences in patient satisfaction. In 2003 Harcourt et al. [7] published a multicenter study reporting that breast reconstruction has a positive influence on anxiety, quality of life (QoL) and self-confidence; however, they reported no significant differences regarding the question of altered body image between patients after mastectomy with and without breast reconstruction, implying that breast reconstruction is not a universal panacea for the emotional and psychological consequences of a mastectomy.

Al-Ghazal et al. [6] reported significant differences in the outcomes concerning satisfaction and psychosocial morbidity (e.g. fear, depression, body image, sexuality and self-confidence) concerning different types of breast cancer surgery. Local wide excision was evaluated to be the best in terms of cosmetic outcomes and psychosocial aspects in their study, followed by breast reconstruction, while mastectomy without reconstruction was associated with lower levels of satisfaction. Accordingly, since skin and nipple-sparing mastectomies (SSM and NSM) have been proven to be oncologically safe, the number of immediate implant-based breast reconstructions has increased, with the best cosmetic outcomes and highest patient satisfaction reported for patients who underwent NSM with immediate reconstruction [8, 9].

We performed a prospective clinical trial investigating patient satisfaction with the cosmetic outcome, the QoL, differences in objective (breast analyzing tool, BAT) and subjective (patients, surgeons, independent breast specialist) evaluations of the cosmetic outcome, and changes in these parameters over time. We hypothesized that patient satisfaction with the cosmetic outcome and the QoL would increase over time, independent of the surgical technique performed.

## Materials and methods

### Patients

Between 2013 and 2014, 61 breast cancer patients were recruited for the study prior to planned surgical treatment for breast cancer. Of the patients 38 (62%) underwent breast-conserving treatment (BCT), while 23 patients (38%) underwent a mastectomy with or without implant-based breast reconstruction (IBBR). In the mastectomy group, 25% underwent modified radical mastectomy (MRM) without breast reconstruction and 3% underwent MRM with delayed IBBR. Lastly, 10% underwent SSM/NSM with immediate IBBR. The *P*-values are purely explanatory and may be interpreted for generating new hypotheses only.

All procedures involving human participants were in accordance with the ethical standards of the institutional and/or national research committee, considering the 1964 Helsinki declaration and its later amendments or comparable ethical standards, and approval for this study was provided by the Institutional Review Board of the Ethics Commission, Medical University Vienna, approval number 073/2010. Informed consent was obtained from all individual participants included in the study.

### Data collection

Data for this study were collected at four individual time points: at baseline (before surgery), on the day of discharge, and 6 and 12 months after surgery.

Clinical data, such as age, tumor type and stage, type of surgery, type of breast reconstruction, and occurrence of complications, were collected from the patients’ medical records.

Digital photographs of the breasts were taken at each time interval. The photos were taken in front of a blue screen. All of them were frontal, including both shoulders and the mammillary fold, without the face. These photographs were then evaluated by the breast surgeon and by an independent breast surgeon using the Harris scale. Furthermore, the photographs were evaluated for breast symmetry using the BAT [10].

Questionnaires were used to assess patient satisfaction and QoL following BCT or mastectomy. A set of questions from the following validated questionnaires were compiled: EORTC (European Organisation for Research and Treatment of Cancer) QLQ (Quality of Life Questionnaire) C30, EORTC QLQ-BR23 (breast cancer module) [11], FBK R‑23 [12], and one question from the Ludwigs-Maximillian University Munich. The questionnaire was then used to evaluate patients’ level of satisfaction with the cosmetic result [13]. These questionnaires were used because of the symptom scales (arm symptoms, breast symptoms, and pain) in the EORTC and because the FBK R‑23 includes items addressing anxiety, psychosomatic disorders, deficit of information, limitations in daily life, and social pressure. The question from the Munich instrument determines patients’ satisfaction with the cosmetic result.

### Evaluating the cosmetic outcome

#### Harris scale

The Harris Scale, a 4-point Likert scale, was used by the surgeon who performed the operation and an independent breast surgeon to evaluate the cosmetic outcome of the treated versus untreated breast after breast cancer surgery [14]. We used the Harris scale because it is a simple and well-established scale with proven utility.

#### Breast analyzing tool (BAT)

The BAT software system calculates a breast symmetry index using digital photographs. The index is calculated by determining the difference in size and shape between breasts (frontal and side view). If the operated breast does not differ in shape and size from the contralateral side, the breasts are considered to be in perfect symmetry. Further information regarding this tool is available in previous literature [10]. We excluded patients who had undergone a total unilateral mastectomy.

#### Statistical analysis

We compared the QoL and cosmetic outcome of patients who received BCT or mastectomy, with or without reconstruction, at four time points (T0 = prior to surgery, T1 = at hospital discharge, T2 = 6 months after surgery, and T3 = 12 months after surgery).

Scores are presented as percent, mean, or median and standard deviation (SD). Wilcoxon signed-rank tests were used to compare the differences between groups and variables at the four time points. A *p*-value <0.05 was considered as statistically significant. All data analyses were conducted using SPSS software (version 21 for Windows, SPSS, Chicago, IL, USA).

## Results

### Patients and follow-up

Of the 61 patients, 57 (93%) completed a baseline (presurgery) questionnaire (T0) and 56 patients (92%) completed the questionnaire on the day of discharge (T1). At the 6‑month follow-up postsurgery (T2), 45 patients (74%) completed the questionnaire and at 12 months post-surgery (T3), 43 patients participated (71%). The total patient drop-out was 29%.

Patients’ characteristics are shown in Table 1. The median age was 59 years (range 37–85 years), 38 patients (62%) underwent breast-conserving surgery and 23 patients (38%) underwent mastectomy. In the mastectomy group, 17 patients (28%) underwent MRM, while 6 patients (10%) underwent SSM/NSM (3 patients SSM; 3 patients NSM) with immediate IBBR and 2 patients (3%) underwent delayed IBBR after MRM, while 15 patients (25%) had no breast reconstruction.

**Table 1:** Patients characteristics

	BCT *n* = 38 (62%)	MRM *n* = 17 (28%)	SSM *n* = 3 (5%)	NSM *n* = 3 (5%)
*Median age *(years; range)	57; 37–76	64; 39–85	55; 52–58	45; 40–52
*Tumor status*				
pTis	8	2	0	0
pT1a	2	1	0	0
pT1b	8	3	1	1
pT1c	14	2	1	2
pT2	6	5	0	0
pT3	0	3	1	0
pT4	0	1	0	0
*Nodal status*				
pN0	26	9	2	3
pN1	3	2	0	0
pN2	0	1	1	0
pN3	1	2	0	0
NA	8	3	0	0
*Histology*				
DCIS	8	2	0	0
IDC	11	5	0	2
ILC	6	0	0	0
IDC + DCIS	13	6	3	1
ILC + DCIS	0	4	0	0
*Reconstruction*				
IBBR	0	0	3	3
Flap reconstruction	0	0	0	0

*BCT* Breast-conserving therapy, *MRM* modified radical mastectomy, *SSM* skin sparing mastectomy, *NSM* nipple sparing mastectomy, *NA* not available, *DCIS* ductal carcinoma in situ, *IDC* invasive ductal carcinoma, *ILC* invasive lobular carcinoma, *IBBR* implant based breast reconstruction

Of the patients 11 (18%) with small tumors (pT1) received mastectomy owing to disease recurrence or a BRCA mutation status. Surgery-associated complications were observed in five patients (8%); one hematoma in the BCT group, one hematoma and one seroma in the mastectomy group and two patients after SSM/NSM with immediate IBBR lost the implants.

### Patient QoL

**Fig. 1:**
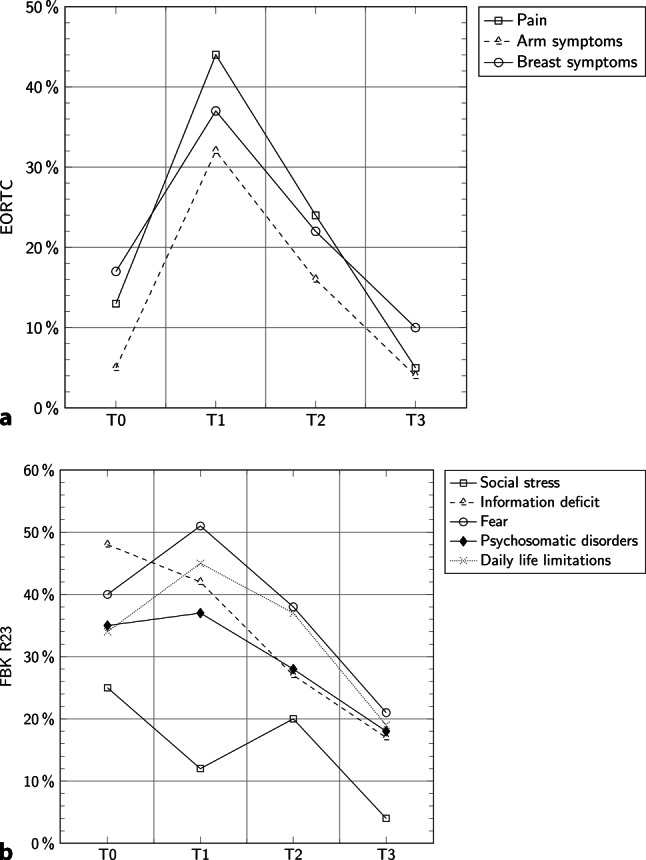
**a** Patient quality of life over time (EORTC), **b** patient quality of life over time (FBK R 23) shown as mean %. Within the EORTC the large number of symptoms after the surgery can be seen, which become lowering over time and were lowest 12 months after. In the FBK R 23 also all limitations were best 12 months after surgery

The percentages given show the questionnaire scores for each symptom or an overall score for the QoL. No significant differences were found between the first and the last visit for arm symptoms (BCT: *p* = 0.500; ME: *p* = 0.892), breast symptoms (BCT: *p* = 0.864; ME: *p* = 0.726), and pain (BCT: *p* = 0.310; ME: *p* = 0.418). These parameters were highest at the time of hospital discharge. Patients’ median QoL was 50% at the time of hospital discharge, the date with the lowest QoL compared to the other time-points (*p* = 0.001), with values of 67% for BCT and 33% for ME. The highest QoL for patients who received BCT was 1 year after surgery, compared to baseline before surgery (BCT: *p* = 0.001, ME: *p* = 0.100). For mean and SD see Table 2. When we investigated if the type of breast surgery had any influence on patients’ QoL, we did not find any statistically significant differences. The information deficit was highest initially (48%) and decreased over time (T3: 17%). At the time of discharge, major issues were fear (51%) and daily life limitations (45%), but these issues also decreased to lower values than at the beginning (fear T3: 21%, information deficit T3: 19%). Fear was significantly lower at T3 than at T0 (*p* = 0.014; Fig. 1).

**Table 2:** Mean and standard deviation of the quality of life at different time points

		Baseline (T0)	Day of discharge (T1)	Postsurgery, 6 months (T2)	Postsurgery, 12 months (T3)
*Total participants*	**–**	57/61 (93%)	55/61 (90%)	44/61 (72%)	42/61 (69%)
*EORTC* *QLQ C 30*	*–*	*Mean score, % (SD)*	*Mean score, % (SD)*	*Mean score, % (SD)*	*Mean score, % (SD)*
Global health status/QoL	Overall QoL	67 (23)	55 (22)	72 (21)	87 (17)
Functional scales	Physical functioning	91 (17)	68 (23)	84 (18)	95 (9)
	Role functioning	91 (19)	56 (36)	73 (27)	90 (18)
	Emotional functioning	62 (23)	61 (27)	67 (23)	79 (21)
	Cognitive functioning	87 (18)	84 (23)	90 (18)	96 (9)
	Social functioning	87 (20)	69 (34)	75 (32)	83 (30)
Symptom scales/items	Fatigue	18 (26)	38 (28)	29 (26)	10 (17)
	Nausea and vomiting	2 (7)	12 (22)	8 (17)	2 (6)
	Pain	13 (21)	43 (29)	24 (23)	5 (13)
	Dyspnea	15 (28)	21 (29)	8 (16)	4 (13)
	Insomnia	28 (32)	41 (34)	24 (29)	14 (22)
	Appetite loss	10 (21)	20 (30)	11 (23)	4 (11)
	Constipation	7 (24)	19 (33)	8 (19)	2 (9)
	Diarrhea	14 (24)	9 (21)	5 (15)	1 (5)
	Financial difficulties	7 (17)	7 (21)	7 (20)	6 (18)
Functional scales	Body image	92 (14)	77 (29)	78 (29)	86 (22)
	Sexual functioning	31 (33)	17 (32)	29 (32)	41 (37)
	Sexual enjoyment	81 (26)	74 (23)	79 (21)	75 (33)
	Future perspective	35 (38)	34 (35)	51 (30)	60 (31)
Symptom scales/items	Systemic therapy side effects	15 (17)	20 (17)	14 (15)	7 (12)
	Breast symptoms	13 (17)	37 (28)	22 (21)	10 (14)
	Arm symptoms	5 (13)	32 (26)	16 (17)	4 (8)
	Upset by hair loss	44 (29)	47 (42)	46 (40)	33 (27)
Patient satisfaction scale	Cosmetic results	N/A	66 (38)	57 (43)	59 (41)
*FBK R 23*	*Baseline (T0)*	*Day of discharge (T1)*	*Postsurgery, 6 months (T2)*	*Postsurgery, 12 months (T3)*	
–	*Mean score, % (SD)*	*Mean score, % (SD)*	*Mean score, % (SD)*	*Mean score, % (SD)*	
Psychosomatic complaints	35 (22)	37 (26)	28 (21)	18 (19)	
Fear	40 (24)	51 (26)	37 (24)	21 (21)	
Information deficits	47 (34)	42 (37)	27 (18)	17 (20)	
Everyday life restrictions	34 (27)	45 (26)	36 (26)	19 (23)	
Social strains	25 (10)	13 (5)	20 (16)	4 (7)	
*Total stress score*	32 (10)	46 (21)	45 (19)	21 (22)	

*EORTC- QLQ* European Organisation of Research and Treatment of Cancer – Quality of Life Questionnaire, *QoL* Quality of Life, *SD* Standard deviation

### Cosmetic results

We took only frontal pictures of the breasts, consistent with the requirements of the BAT, which were used to measure breast symmetry.

**Fig. 2:**
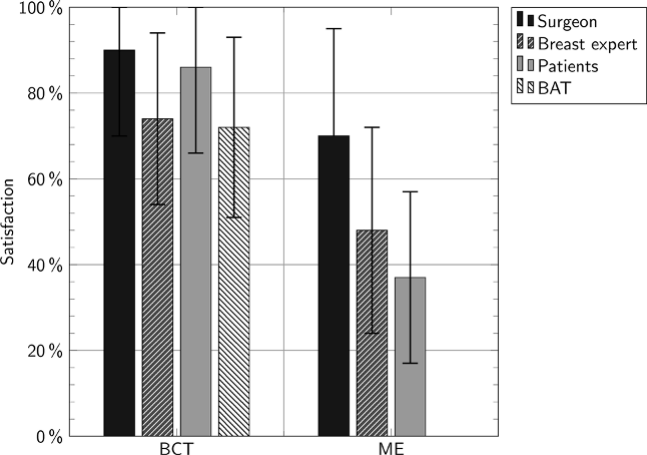
Satisfaction with the cosmetic result (mean values and SD). The difference of the satisfaction with the cosmetic result within BCT and ME is shown. Surgeons were more satisfied with the cosmetic result than patients and the breast specialist. Patients with ME are not as satisfied as patients with BAT

The mean satisfaction of the surgeon with the cosmetic result was high (mean of 83% on the Harris scale, 90% for BCT, and 70% for ME). The satisfaction of the specialist who was not involved in the surgery of the individual patient was lower (mean of 64% on the Harris scale, 74% for BCT, and 48% for ME; *p* = 0.001). A frequency table of the Harris scale from the surgeons and the specialist is shown in Table 3. The satisfaction of the patients was very different between those who received BCT (mean 86%) and ME (mean 37%). The BAT score requires a nipple and therefore it was not possible for ME. The mean for BCT was 72% (Fig. 2).

**Table 3:** Frequency table of the Harris scale for surgeons and the specialist

	Excellent	Good	Fair	Poor	Missing data
Surgeon	34	10	8	4	3
Specialist	10	23	17	9	0

The BAT score was lowest (24%) prior to surgery, indicating that the breasts were the most symmetrical at this time point. Breast asymmetry was highest on the day of discharge. This difference was statistically significant (*p* = 0.049). Over time, the BAT score improved and almost returned to the baseline score at 12 months postsurgery, as did the satisfaction of patients who underwent BCT (*p* = 0.049).

**Fig. 3:**
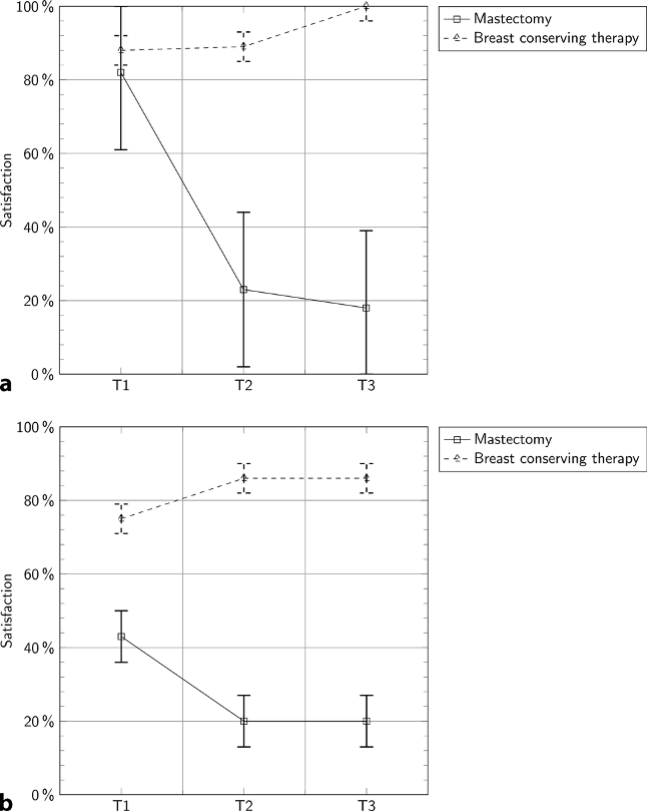
**a** Patients <65 years who were satisfied or very satisfied with surgery outcome; **b** Patients >65 years who were satisfied or very satisfied with surgery outcome shown as mean % and SD. Patients with BAT become more satisfied with the cosmetic result over time, but a difference between ages can be seen

At the time of hospital discharge (T1) 57% (BCT: 62%, ME: 47%) of all patients were generally satisfied with the cosmetic result, while 18% (BCT: 11%, ME: 31%) of patients were dissatisfied; the remainder did not answer this question. Six months after surgery (T2) 58% (BCT: 79%, ME: 19%) of all patients were satisfied and 38% (BCT: 14%, ME: 81%) were dissatisfied with the cosmetic result and 12 months after surgery (T3) 58% (BCT: 85%, ME: 13%) were satisfied, while 28% (BCT: 7%, ME: 63%) were dissatisfied. The mean age of patients who were “not at all” or “only a little” happy with the cosmetic result was 64 years (BCT: 65 years, ME: 58 years). Those who were “quite a bit” or “very much” happy were on average 58 years old (BCT: 57 years, ME: 65 years). Of the patients 41 (67%) were under 65 years old. We found no significant difference in stage, tumor type, or method of operation. Patients were especially dissatisfied with the cosmetic outcome on the day of discharge compared to baseline (*p* = 0.024). Furthermore, satisfaction with the cosmetic result significantly decreased after discharge in patients younger than 65 years who underwent mastectomy (*p* = 0.049), but not in patients over 65 years who underwent mastectomy (Fig. 3).

**Fig. 4:**
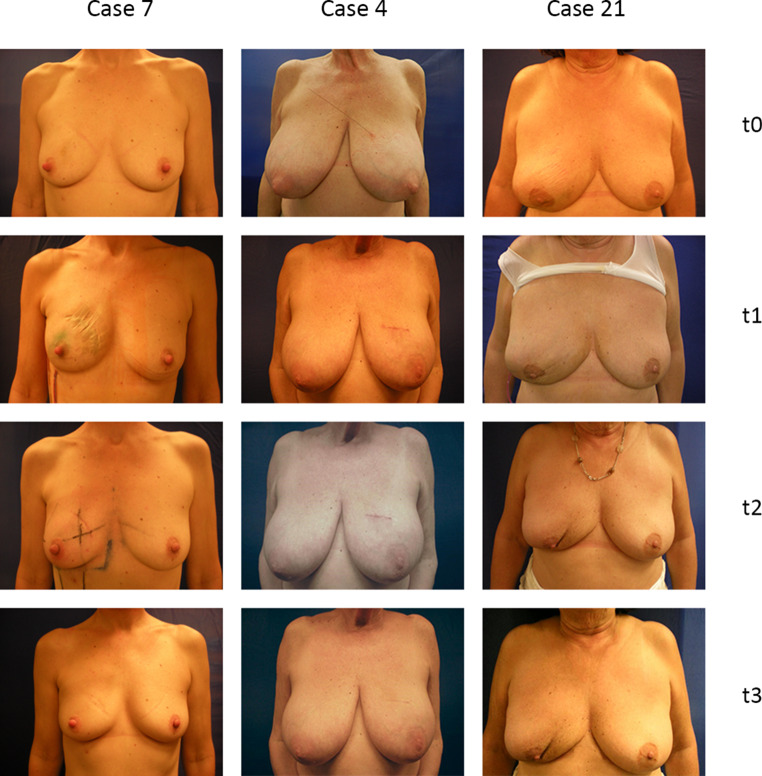
Examples of good and poor cosmetic outcome. *Case 7*: invasive ductal carcinoma, pT1b, breast conserving therapy, BAT score 2, specialist and surgeon evaluated as excellent outcome Harris scale 4. *Case 4*: invasive ductal carcinoma, pT1c, breast conserving therapy, BAT score 4, specialist and surgeon evaluated as excellent outcome Harris scale 4. *Case 21*: invasive ductal carcinoma, pT1b, breast conserving therapy, BAT score 6.5, specialist and surgeon evauated as poor outcome Harris scale 2

Fig. 4 shows examples of a poor cosmetic outcome (case 21; BAT score 6.5), a median example on the basis of the BAT score (case 4; BAT score: 4), and a good cosmetic outcome (case 7; BAT score: 2). The surgeon and the specialist evaluated cases 7 and 4 as an excellent cosmetic outcome (Harris scale: 4), while case 21 was evaluated as poor (Harris scale: 2).

In mastectomy patients, the satisfaction of the cosmetic result was higher when the resected volume was lower (*p* = 0.023) We did not find the same phenomenon in patients who received BCT (*p* = 0.065). The resected volume of a mastectomy was linear to the cup size of the patients.

## Discussion

Our study investigated the impact of breast surgery on patients’ QoL and found a significant improvement in patients’ QoL and cosmetic outcome over time. This is consistent with previous findings showing that most long-term survivors of breast cancer ultimately reach QoL levels comparable to healthy controls [15, 16].

The improvement in the overall QoL over time may indicate that patients had more time to come to terms with the severe diagnosis of breast cancer. Most of the study participants completed the treatment by their third or fourth visit. This was reflected in the observed increase in emotional functioning, which has also been reported previously [7, 17]. We found a significant decrease in daily life limitations 12 months after surgery. We suggest that this is because of the anxiety and frequent examinations before the operation, so that the patients felt limited in their social life.

Our study also showed that older patients are less satisfied with the cosmetic outcome than younger women immediately after surgery, but not as time progresses. The groups also did not differ in stage, tumor type, or method of operation. The lower satisfaction might be due to age-based self-perception differences or other reasons, which would require more exploration in future studies, because of the small sample size.

Breast asymmetry improved over time and almost returned to the baseline scores 1 year after surgery. As shown in our previous study, breast symmetry has no significant impact on QoL in breast cancer survivors [18], which has since been validated in a larger study [19]. Furthermore, we did not find any association between the type of breast surgery and patients’ QoL or their satisfaction with the cosmetic result. This could be because patients who receive BCT in our department automatically receive oncoplastic surgery to reconstruct the defect. In most cases, this is a simple glandular rotation. Between patients undergoing ME with or without reconstruction, we did not find any differences in QoL and satisfaction with the cosmetic outcome. The small study population, especially in the reconstructed group, could be the reason.

Our study was limited by the decrease in patient participation over time (from 93% to 71%). This is not uncommon and is similar to other studies [20]. It could have been because of a bias that unsatisfied patients changed the institution. Since our institution is a large hospital, many patients come for surgical treatment and change to a smaller, local hospital for further treatment. Higher participation was observed in our study at the two initial time intervals, likely because patients were admitted for the scheduled surgery and were still hospitalized after the procedure. At the later time points, a decline in participation could be due to diminishing motivation or interest as well as difficulty in transportation, especially for older patients. We only took frontal pictures of the breasts since this approach was standardized and easy to perform. In future studies, pictures should ideally be taken from multiple different standardized planes. Lastly, the study is also limited because of the small sample size and the variety of operation types, which reduced the size of the subgroups. An endpoint was difficult to define because all scores were subjective, although we improved objectivity by using the BAT.

In conclusion, our study shows that the QoL of breast cancer patients is significantly affected by surgical treatment, but only immediately after breast surgery. Older patients were less satisfied with the cosmetic result directly after surgery, which emphasizes the importance of integrating possible postsurgery cosmetic outcomes into the preoperative information for older patients, to the same extent as for younger patients.

Lower resected volume is associated with higher satisfaction of patients in mastectomy. Perhaps this phenomenon could be seen because of the lower body mass index (BMI) of these patients and the associated lower number of side effects [21].
